# Studies on Carcinogenic and Toxic Effects of Ochratoxin A in Chicks 

**DOI:** 10.3390/toxins2040649

**Published:** 2010-04-12

**Authors:** Stoycho D. Stoev

**Affiliations:** Department of General and Clinical Pathology, Faculty of Veterinary Medicine, Trakia University, Students campus, 6000 Stara Zagora, Bulgaria; Email: stoev@uni-sz.bg; Tel.: +359-42-670-540; Fax: +359-42-670-624.

**Keywords:** ochratoxin A, phenylalanine, carcinogenic effect, chicks, pathology

## Abstract

Carcinogenic/toxic effects of ochratoxin A (OTA) in various internal organs of Plymouth Rock chicks were determined. The number of OTA-induced neoplasms was similar in chicks given 25 ppm L-β-phenylalanine (PHE) in addition to 5 ppm OTA compared to chicks given only 5 ppm OTA, which showed that PHE cannot be used as a real protector against the carcinogenic or toxic effects of OTA in chicks. OTA was found to provoke strong degenerative changes in liver and kidneys, degenerative changes and depletion of cells in lymphoid organs, oedematous and degenerative changes in the brain, muscular haemorrhages and fatty changes in the bone marrow. The target organs for carcinogenic effect of OTA in chicks were found to be kidneys and liver.

## 1. Introduction

Ochratoxin A (OTA) is a mycotoxin widely encountered in animal feeds and human foods. The most obvious effects of OTA-contaminated feed on chicks are reported to be reduced rates of weight gain [1,2,3], decreased egg production [4], immunosuppressive effects [2] and increased mortality [5]. In addition, there are many other reports on various carcinogenic effects of OTA, but in these attention is usually focussed on mice or rats [6,7,8] and there is a lack of evidence with regard to carcinogenic effects of OTA on chicks. It is also known that the IARC has classified OTA as a possible human carcinogen (group 2B) based on sufficient evidence for OTA-carcinogenicity obtained from various animal experiments [9].

Feed supplements are often studied with regard to their potential to ensure safely utilisation of some mycotoxin-contaminated feeds. Haazele *et al.* [4] reported that ascorbic acid supplementation in laying hen diets can partially reduce the toxic effects of nephrotoxic mycotoxin OTA. Some authors suggest that part of the toxic effect of OTA is due to the structural homology with phenylalanine (PHE), resulting in an inhibition of protein synthesis due to a competition for the specific t-RNA [10,11]. However, this effect has been demonstrated only in bacteria and yeast [10,11], but has never been seen in animals. Nevertheless, a protective effect of PHE administration has been reported by some authors [12]. Therefore, PHE was used in the present experiment in other to evaluate its possible protective effect against OTA. 

This experiment could be considered as an extension of our former short term studies on the toxic effects of OTA on various internal organs, biochemical indices and body weight gain in stock chicks, published in *Toxicology Letters* [3]. The main aim of the present study was to evaluate the possible long term toxic or carcinogenic effects of OTA and the possible protective effects of PHE. 

## 2. Materials and Methods

### 2.1. OTA production

A strain of *Aspergillus ochraceus* (isolate D2306, as used by Tapia and Seawright [13], and Stoev *et al.* [3]) was grown on sterilised shredded wheat (40 g) in 500 mL conical flasks moistened by a 40% (v/w) addition of sterile water and incubated on a rotary shaker at 27 °C for 2 weeks [14]. The brown granular product was then sterilised at 80 °C for 1 hour and stored at −20 °C until the study. A sample was analysed by HPLC with diode array detection for OTA and found to contain ~2 mg/g of this compound and a relatively small amount of its dechloro-analogue ochratoxin B [14]. No other mycotoxins were produced in this solid substrate fermentation process and the necessary dilution by approximately 10^3^ when homogenised into chick ration made only a minimal addition of other components of the moulded shredded wheat substrate.

### 2.2. Experimental design

Specific pathogen-free chicks (Plymouth Rock) were purchased at two-weeks of age, housed in wire floor cages (two chicks in each cage with more than 500 cm^2^ per chick at the beginning of experiment; one chick in each cage with more than 1,000 cm^2^ per chick at later stages of sexual maturity) with continuous infra-red lighting at a temperature suitable for their age. Commercially prepared complete standard feed (Smesler, Stara Zagora, Bulgaria) suitable for the breed and age of the chicks according to the accepted standards and regulations of the country, which contained the defined concentrations of OTA with or without PHE supplementation, supposed to protect against the toxic effects of OTA, were available *ad libitum* during the whole experimental period as described in Table 1. The chicks were grouped in two experimental and one control group (10 chicks in each group—five males and five females) as shown in Table 1.

The L-β PHE (Finomvegyszergyar, Budapest, Hungary) was used in 5:1 molar ratio with regard to OTA as the higher doses (above 10:1 molar ratio) provide only a slight protection against OTA, because of increasing the absorption of OTA from the stomach and intestine. In addition, the higher supplementation of pure phenylalanine in diets contaminated with OTA also tended to create an amino acid imbalance, which reduced b. w. gain and feed conversion efficiencies [15,16].

**Table 1:** Amount of ochratoxin A (OTA) and PHE in experimental feeds of chicks.

Group	Added OTA in feed (ppm - mg/kg)	Added PHE in feed
Control	none	none
1	5	none
2	5	0.0025% (0.025 g/kg feed - 25 ppm) L-β PHE

OTA—ochratoxin A; PHE—phenylalanine.

The Trakia University Animal Care Ethic Committee approved the study protocol and chicks were housed, maintained and slaughtered in accordance with the Bulgarian Welfare Regulations.

### 2.3. Measurements of tumours incidents

All chicks were examined for the existence of various neoplastic tissues at the end of the two years experimental period, when the same were slaughtered. Such examination was also made in all chicks, which were dead during the experiment. Non-parametric Mann–Whitney and Student’s *t*-test were used to estimate significant differences between tumour incidences in experimental and control groups. 

### 2.4. Histological examination

Materials for histological examination were taken at slaughter time (at about 24-months age) or after the death of the chicks from kidneys, liver, heart, thymus, bursa Fabricii, spleen, intestine, cerebellum, brain, medulla and bone marrow as well as from various neoplastic tissues. The same were fixed in 10% neutral buffered formalin or processed for freezing microtome. The freezing materials were stained with Sudan III for proving the fat. The fixed tissues were processed for paraffin embedding, sectioned at 6 µm and stained with haematoxylin-eosin. Periodic acid-Schiff (PAS) stain was also used for proving of lipoproteid, glycoproteid or mucoproteid substances in various tissues and cell components, and especially for proving the thickening of basement tubular membranes (with lipoprotein structure). Some materials were stained according to Weigert iron haematoxylin for proving the presence or absence of fibrin in various cyst formations.

## 3. Results

### 3.1. Clinical observation and gross pathology

The most obvious clinical signs as weakness or dullness, goose plumage, transient diarrhoea and growth depression were seen in all chicks from the groups treated with OTA, but the same were less pronounced in chicks supplemented additionally with PHE. Various nervous symptoms such as torticollis, emprosthotonus, lurch, reeling gait (staggering step) and tremor were seen in different degree in all chicks treated with OTA alone, but only in half of the chicks treated with PHE in addition to OTA. Some of these clinical signs as weakness or dullness were seen just after 2–3 weeks of feeding the contaminated diet, whereas other signs as nervous symptoms appeared after 10–15 weeks, but were more pronounced at the end of the first year of the experiment.

**Figure 1:**
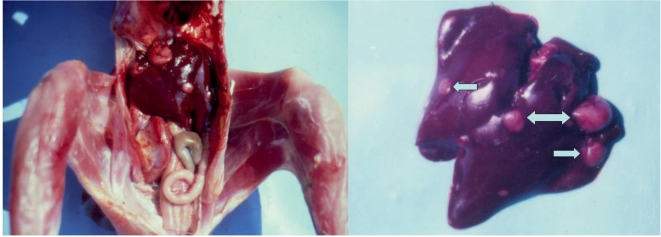
Adenocarcinoma in the liver of male chick of group 1 exposed to 5 ppm OTA *via* the feed, which died at the end of the 10th month of the experiment. Large grey-white neoplastic foci are seen in the liver and protruded significantly above its surface.

At the end of the 10th month of the experiment one male chick from group 1 was dead. Several large grey-white neoplastic spots were seen in the diaphragmatic and abdominal surfaces of the liver in the same chick (Figure 1), which were subsequently diagnosed as adenocarcinoma. The same protruded significantly above the liver surface (Table 2).

**Table 2:** Kind of neoplasm and time of establishment in various tissue or organs of male or female chicks exposed to OTA and treated with or without PHE *via* the ingested feeds.

Group	OTA in feed (ppm-mg/kg)	PHE in feed	Kind of neoplasm	Benign or malignant	Time of establishment	Tissues or organs	Sex of chick
1	5	none	adenocarcinoma	malignant	10^th^ month	liver	male
1	5	none	lymphosarcoma	malignant	18^th^ month	kidney	male
1	5	none	carcinoma	malignant	20^th^ month	ureters	male
1	5	none	cystic adenoma	benign	24^th^ month	kidneys	male
1	5	none	cystic adenoma	benign	24^th^ month	kidneys	female
2	5	L-b PHE	adenocarcinoma	malignant	19^th^ month	kidney	female
2	5	L-b PHE	carcinoma	malignant	21^st^ month	liver, spleen	female
2	5	L-b PHE	rabdomyoma	benign	24^th^ month	breast muscle	female

OTA—ochratoxin A, PHE—phenylalanine.

**Figure 2:**
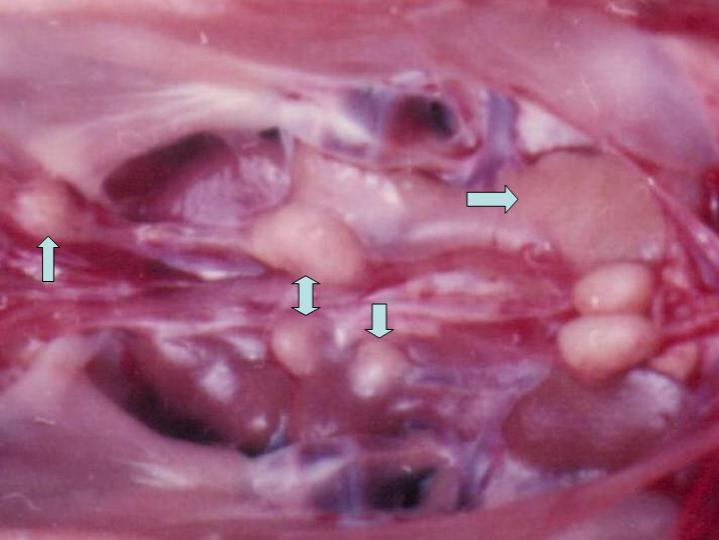
Carcinoma in the region of ureters of male chick of group 1 exposed to 5 ppm OTA *via* the feed, which died at the end of the 20th month of the experiment. Large grey-white neoplastic foci are seen along the ureters and protruded significantly above its surface.

**Figure 3:**
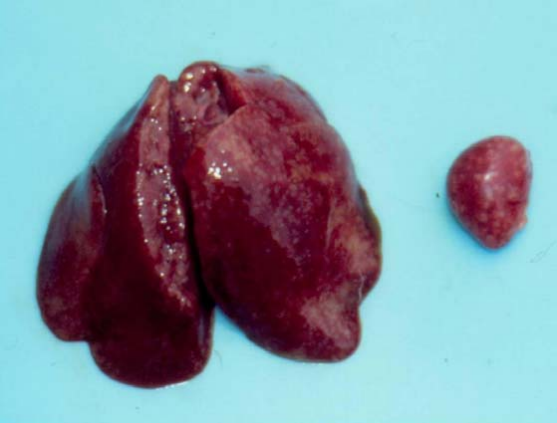
Carcinoma in the liver and spleen of female chick of group 2 exposed to 5 ppm OTA and 25 ppm PHE *via* the feed, which died at the end of the 21st month of the experiment. Small grey-white neoplastic foci are seen on the surface of liver and spleen.

Four chicks died between the 12th and 24th month from the beginning of the experiment, because of development of different kind of malignant tumours diagnosed as lymphosarcoma in kidney (chick from group 1), adenocarcinoma in kidney (chick from group 2), carcinoma in the region of ureters (chick of group 1—Figure 2), carcinoma in the liver and spleen (chick of group 2—Figure 3) at the time of pathomorphological examination (Table 2).

**Figure 4:**
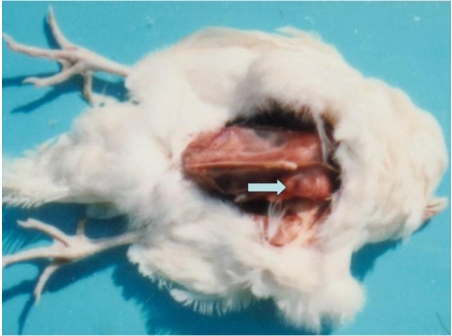
Rabdomyoma in the breast muscle of female chick of group 2 exposed to 5 ppm OTA and 25 ppm PHE *via* the feed, which was slaughtered at the end of the 24th month of the experiment. Large neoplasia in the region of breast muscle, which protruded significantly above the surface.

Two more benign tumours were found at the slaughter time (24 months from the beginning of experiment). The same were defined microscopically as rhabdomyoma in the breast muscles (chick from group 2—Figure 4) or adenomas in the kidney (Table 2). There were no any neoplasms in the chicks from control group.

The comparison between experimental and control groups revealed a significant increase of tumour incidents in the chicks exposed to OTA towards the controls, but no significant protective effect was found in regard to PHE.

Gross pathology examination at the end of the two-year experimental period revealed that all chicks from OTA-treated group were emaciated and their bones were sometimes thin or soft. There were some musclular haemorrhages and slight catarrhal enteritis in the same chicks. In addition, slight subcutaneous oedema was found in a few chicks from the same group. Kidneys and liver in the same experimental chicks as well as in the chicks supplemented with PHE in addition to OTA were slightly paler than in the chick of control group, whereas their gall bladders were enlarged and full of bile. A different extent of contraction and diminished size of kidneys were also observed in OTA-treated chicks and in lesser extent in chicks additionally supplemented with PHE. Some parts of the bone marrow in OTA-treated chicks were with grey-white colour, whereas others showed a preservation of the normal red colour. The meninges of the same chicks were slightly hyperaemic. There were no gross pathology changes in the chicks from control group.

### 3.2. Histopathology

Pathomorphological investigation of kidneys showed degenerative changes (granular or hydropic degeneration, karyomegaly, karyopyknosis or karyorrhexis) in epithelial cells of the proximal convoluted tubules, cystiform dilatation of the lumen of some tubules, tubular atrophy in other tubules, mononuclear proliferation in the mucosa of ureters, and moderate proliferation of connective tissue and mononuclear cells in the renal interstitium, seen mainly in chicks of OTA-treated group and in lesser extent in chicks of group additionally treated with PHE. Different degree of congestion of peritubular capillaries, oedema, activation and proliferation of capillary- or vascular endothelium and vascular adventitial cells of kidneys were also seen in the same both groups. The existence of various kinds of distended tubules with atypical epithelial cells was also a frequent finding in the kidneys of the same chicks.

**Figure 5:**
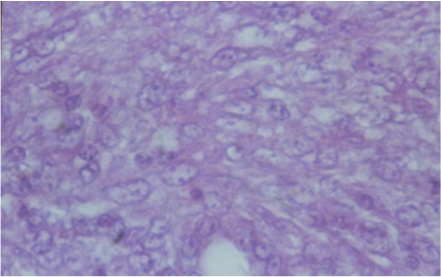
Photomicrograph of the secondary metastases in the kidneys coming from the initial carcinoma in the region of ureters of male chick of group 1 exposed to 5 ppm OTA *via* the feed, which died at the end of the 20th month of the experiment. Infiltrative or destructive growth and pronounced polymorphism of neoplastic cells, showing large hypochromatic or polychromatic nuclei. Carcinomatous and single cyst-like formations in the kidneys. HE × 400.

**Figure 6:**
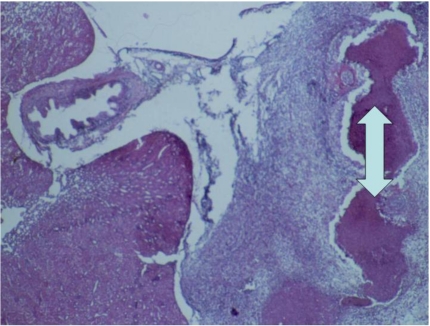
Photomicrograph of the carcinoma in the region of ureters of male chick of group 1 exposed to 5 ppm OTA *via* the feed, which died at the end of the 20th month of the experiment. Large necroses among the neoplastic tissue. HE × 100.

In the case of the initial solid carcinoma in the region of ureters (Figure 2), many secondary metastases were also found in the kidneys (Figure 5). These metastatic spots showed infiltrative or destructive growth within adjacent parenchyma and pronounced polymorphism of neoplastic cells, which had large hypochromatic or polychromatic nuclei, and many irregular mitoses. Large necroses were also found among the neoplastic tissue (Figure 6). In a few places these solid carcinomatous formations changed into small cyst-like formations lined with several layers neoplastic cells (adenocarcinoma).

The adenocarcinoma of kidneys showed cyst-like formations lined with several layers of atypical cells with polymorphism and large hypochromatic or polychromatic nuclei. Several metastases in the lung were also found in the same chick.

The lymphosarcoma of kidneys was accompanied by glomerular or tubular atrophy and large necroses among the neoplastic tissue.

The cystic adenomas of kidney had small sizes without destructive growth and penetration in the adjacent parenchyma and were constituted of cyst-like formations lined with one layer neoplastic cells without polymorphism or metastases in the adjacent parenchyma.

In the liver, cloudy swelling, granular degeneration and rarely fatty changes of the hepatic cells were seen in the chicks of OTA-treated group, but the same changes were less obvious in the chicks supplemented with PHE. Activation of capillary endothelium and Kupffer's cells, hyperaemia, pericapillary oedema, perivascular mononuclear cell infiltration, proliferation of connective tissue and bile ducts were also noticed, mainly in the chicks of OTA-treated group.

**Figure 7:**
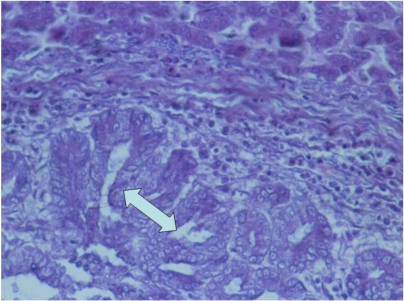
Photomicrograph of the adenocarcinoma in the liver of male chick of group 1 exposed to 5 ppm OTA *via* the feed, which died at the end of the 10th month of the experiment. Neoplastic cells having large hypochromatic or polychromatic nuclei and giving cyst-like formations lined with several layers of neoplastic cells. HE × 300.

In the case of adenocarcinoma in the liver, neoplastic tissue showed infiltrative or destructive growth within adjacent parenchyma and was constituted of cyst-like formations lined with several layers of neoplastic cells (Figure 7) having polymorphism and large hypochromatic or polychromatic nuclei along with small quantity of cytoplasm and lots of irregular mitoses. In some places these cyst-like formations were replaced by solid carcinomatous formations having an increased polymorphism, large quantity of irregular mitoses, and lots of polychromatic nuclei. A small quantity of secret with desquamated epithelial cells was seen in the lumen of some cyst-like formations. Large necroses were often seen among the neoplastic tissue. Strong degenerative changes, in addition to slight polymorphism of hepatic cell, including polychromatic nuclei, were found in the parenchyma adjacent to neoplastic tissue. Intranuclear inclusions were sometimes seen in some of the neoplastic cells or in the hepatic cells adjacent to neoplastic tissue. Different degree of karyomegaly was also observed in some hepatic cells adjacent to neoplastic areas. Slight to moderate hyperaemia of capillaries or vessels was seen in the same areas. 

**Figure 8:**
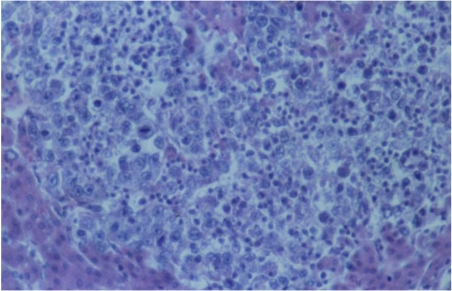
Photomicrograph of the carcinoma in the liver of female chick of group 2 exposed to 5 ppm OTA and 25 ppm PHE via the feed, which died at the end of the 21st month of the experiment. Small carcinomatous formations of neoplastic cells having an increased polymorphism, large quantity of irregular mitoses, lots of polychromatic nuclei and destructive growth within the adjacent parenchyma. HE × 300.

The carcinoma of liver and spleen showed lots of similar neoplastic spots in both liver and spleen. Neoplastic carcinoma cells had an increased polymorphism, large quantity of irregular mitoses, lots of polychromatic nuclei and small necroses among neoplastic tissue (Figure 8). Carcinoma cells showed a typical strong infiltrative or destructive growth within adjacent parenchyma.

In the thymus and bursa of Fabricius, a depletion of lymphoid cells, atrophy, necroses or cyst formations in the lymph tissue were seen in the chicks of both OTA-treated groups. In the chicks of control group, only atrophy of lymph tissue or lymph follicles as well as proliferation of connective tissue around the follicles, corresponding to the involution of the same organs, were seen. A cellular depletion or degenerative changes were also found in the white pulp and germinal centres of the spleen in the chicks of the both OTA-treated groups. 

Slight to moderate degenerative changes or depletion of lymphoid cells were also seen in the lymph follicles of intestinal mucosa in the chicks of both OTA-treated groups. A mononuclear and heterophilic cell infiltration was often seen in the lamina propria of intestinal mucosa of the same chicks. Small foci of focal necrosis and loss of surface epithelium accompanied by degenerative changes in the glandular epithelium were found only in the intestinal mucosa of chicks treated with OTA alone.

In the heart, granular or vacuolar degeneration, lytic changes or irregular staining as a result of the increased eosinophilia of some myofibrils as well as activation of capillary endothelium and perivascular oedema were mainly seen in the chicks treated only with OTA, while the same changes were less pronounced in the chicks additionally supplemented with PHE.

In the case of rabdomyoma in the breast muscle, the neoplastic tissue was constituted of myofibrile-like but not striated muscle cells showing a much paler colour compared to the ordinary myofibrils. Destructive growth or metastases in the adjacent areas were not observed.

In the lung, only perivascular or peribronchial mononuclear cell infiltration was found in some chicks of both groups treated with OTA. 

The pericapillary or pericellular oedema and lytic changes in neurons and glia cells were the most obvious pathological changes in the brain of all OTA-treated chicks. Activation and proliferation of capillary- or vascular endothelium and vascular adventitial cells were seen in some vessels of the brain in the chicks of the group treated with OTA alone. Small foci of proliferation of glia cells around some vessels or necrotic neurons were rarely seen in OTA-treated chicks.

Similar oedematous and degenerative changes were also found in the cerebellum, mainly in the region of the Purkinje's cells, in the chicks of both OTA-treated groups. Well visible oedematous changes in the white substance of cerebellum were also found in the chicks treated with OTA alone.

In the medulla, lysis and pyknosis of tigroid substance of some neurons were seen mainly in the lumbosacral region in the chicks of the group treated with OTA alone.

In the bone marrow, cell depletion and fatty changes were only noticed in the chicks of both experimental groups.

No pathological changes or neoplasms were seen in the internal organs of the control chicks. Only a natural involution of the bursa of Fabricius and thymus were found in the same chicks.

## 4. Discussion

The neoplasms (tumours) and pathological changes observed in experimental chicks are considered to be due to OTA-exposure, because such pathological changes or tumours were not observed in the control chicks and no infections or other diseases were seen in any of the experimental chicks.

Histopathological changes seen in both experimental groups of chicks correspond well to the clinical signs observed. The strong degenerative changes seen in the epithelial cells of the kidneys or the liver could be explained by the route of elimination of OTA via the kidneys and the liver, having in mind, enterohepatic recirculation and hepatobiliar way of excretion of OTA [17]. In such a way this mycotoxin can exert a direct toxic or carcinogenic effect on these organs [1,2]. That could explain the main localization of various neoplasms in the both organs. The most obvious lesions in the kidneys were present in the epithelium of proximal tubules in chicks of all experimental groups, whereas the proliferative changes in the interstitium could be considered as a sequence of the strong degenerative changes in the epithelium and vessels in later stages of OTA exposure. Oedematous changes in the brain and cerebellum in addition to various subcutaneous haemorrhages might be due to the vascular damages induced by OTA [2,3,18,19], which are considered to be typical for later stages of this nephropathy. A disturbance of blood clotting due to a reduction in the concentration of fibrinogen in the blood and an increase in the prothrombin time, found in OTA-treated animals [20,21], could also contribute to the haemorrhages observed.

The observed karyomegaly in hepatic cells adjacent to neoplastic tissue should be considered as an indicator of the partial regenerative activity of parenchyma in the damaged area [22]. On the other hand, karyomegaly and especially aneuploidy or polyploidy in the nuclei, in addition to cellular polymorphism, usually are considered as pre-carcinogenic state and good indicators for future carcinogenic transformations of the cells, when the same are further exposed to target carcinogens. Similar pathological changes were recently observed in rats fed OTA [23,24]. 

On the other hand, various kinds of distended tubules with atypical epithelial cells in the kidneys could be considered as pre-neoplastic kidney lesions in the affected kidneys [25], whereas benign adenomas in kidneys are usually the initial stage of development of malignant tumours in kidneys [26].

Several line of evidences indicated that carcinogenic effect of OTA is correlated to its genotoxic effect reflected by DNA-adducts formation [8,27,28]. It is also important to mention, that the genotoxic effect in various animals has been observed in significantly low OTA-intake, similar to that observed in humans. The more frequent neoplasms in the male chicks in comparison to the female is well corresponding to the studies of Bendele *et al.* [29], Castegnaro *et al.* [7] and Pfohl-Leszkowicz *et al.* [30,31], who found a similar trend among the male mice or rats exposed to diet containing OTA. According to Pfohl-Leszkowicz *et al.* [30,32] this sex-specific response could be due to the difference in some drag-metabolizing enzymes that convert OTA into reactive intermediates. However, in chicks given PHE, such a higher sensitivity in male chicks to carcinogenic effect of OTA was not observed in the present study.

The circumstance, that some of the tumours were situated in the liver, in contrast to other farm animals, correspond well to the fact that a large amount of OTA in chicks is also excreted *via* the liver and not only via the kidneys, which may explain the strong degenerative changes found in livers of OTA-treated chicks. A similar finding of OTA-induced tumours, including liver neoplasms, is also characteristic for mice or rats [6,26]. Unfortunately, there is not information available about similar kind of experiments among chicks and further comparison would not be able to be done. 

The found carcinoma in the ureters of chick is really interesting findings, which is in good agreement with the high frequency of carcinoma in the renal pelvis, ureters and urinary bladder found in patients suffering from Balkan Endemic Nephropathy [33,34], a disease supposed to be caused by nephrotoxic mycotoxins and mainly OTA. Such carcinomas have not been seen in any kind of farm animals so far. In this regard, it is important to mention that some tumours in kidneys and bladders from patients living in areas associated with Balkan Endemic Nephropathy have been found to contain DNA adducts similar to those obtained from the kidneys of mice exposed to OTA [35,36,37], which suggests about the possible role of OTA in the development of these tumours of the urinary tract.

It has been found that OTA decreases natural killer cell activity by the specific inhibition of endogenous interferon levels [38]. As natural killer cells are involved in the destruction of tumour cells, the ability of OTA to modulate the activity of these cells might contribute to its capacity to induce renal and hepatic carcinomas [38].

In field conditions OTA is only part of a complex array of mould metabolites, such as penicillic acid, fumonisins, citrinin, *etc.* [39,40], which have also carcinogenic effects [41,42,43,44] and may interact in this dimension with OTA, which is also proven carcinogen. Moreover, fumonisin B1 was found to have a pronounced nephrotoxic effect on animal kidneys [45,46,47,48], which can be additive to nephrotoxic effect of OTA [37,39,40]. All these circumstances suggest the importance of the relatively lower doses of OTA that commercial chicks may encounter in some feeds.

The utilization of various means to reduce OTA-toxicity (as PHE supplementation) supposes a good understanding of the possible mechanisms of OTA-toxicity: an inhibition of enzymes involved in phenylalanine metabolism (including phenylalanyl transferase and phenylalanine hydroxylase), an effect on lipid peroxidation, an effect on mitochondrial function (inhibition of ATP production), an ability to provoke a dose-related increase in sister chromatid exchange (SCE) in Chinese hamster ovary cells (CHO) and to cause DNA single-strand break [25,26,49,50]. This approach includes an addition of PHE and protein to the diet, because PHE moiety of OTA could competitively inhibits at least two enzymes as has been reported in bacteria or yeast: phenylalanyl-tRNA synthetase and phenylalanine hydroxylase, resulting in reduced protein synthesis and altered rates of tyrosine production from PHE. In such a way, co-administration of PHE with OTA may reduce OTA-induced inhibition of protein synthesis [51,52]. In this regard, a slight protective effect of PHE was previously found against OTA-provoked immunosuppression in humoral immune response that was supposed to be due to improvement of protein synthesis, which is usually disturbed by OTA and subsequently, improvement of OTA-induced delay of the division of the cells of the immune system [3]. 

It was surprising however, that the protective effect of PHE in the present experiment was significantly lower than our expectation. This could be explained by the circumstance that PHE was found to increase the absorption of OTA from the stomach and intestine as well as the gastrointestinal transit of OTA resulting in fourfold up to eightfold higher levels of OTA in serum, as has been reported in some experiments with mice [17]. On the other hand the main mechanisms of OTA-toxicity in our case may not involve the inhibition of phenylalanine metabolizing enzymes. It has been demonstrated recently that carcinogenic and nephrotoxic processes induced by OTA are driven by two independent pathways: one involving a mainly oxidative pathway and another involving the biotranformation into gentoxic OTA derivatives [31].

## 5. Conclusions

In this study we demonstrated that OTA induces significant pathomorphological damages in kidneys, liver, lymphoid organs, brain and bone marrow of chicks, as well as some tumours in kidneys, ureters, liver, spleen and muscles. A significant protection of PHE against the carcinogenic or toxic effects of OTA in chicks was not observed in the present experiment. 
